# Bullying Victims’ Perceived Social Support and Psychological Health and Prosocial Behavior: A Latent Profile Analysis

**DOI:** 10.1007/s10964-024-01954-3

**Published:** 2024-03-01

**Authors:** Yanghua (Felicia) Huang, Harold Chui

**Affiliations:** Department of Educational Psychology, The Chinese University of Hong Kong, Hong Kong SAR, China

**Keywords:** School bullying, Social support, Psychological health, Prosocial behavior, Person-centered approach, Latent profile analysis

## Abstract

The literature on school bullying and perceived social support primarily relies on variable-centered approaches, investigating the independent effects of individual sources of social support. However, victims of school bullying perceive different combinations of levels of social support from multiple sources. Hence, there lacks a holistic person-centered examination of the joint effects of multiple sources of social support. The study surveyed 915 bullying victims (51.9% boys, 48.1% girls; *M*age = 13.52, *SD* = 0.75). Latent profile analysis identified five profiles of social support across five sources (i.e., parents, teachers, classmates, close friends, and online-only friends): *online-offline supported* adolescents, *offline supported* adolescents, *moderately supported* adolescents, *close friend supported* adolescents, and *unsupported*. The five social support profiles were associated differently with bullying victims’ demographic characteristics (i.e., age, gender, and socioeconomic status), frequency of victimization, psychological health (i.e., subjective well-being, depression, and anxiety), and prosocial behavior. The findings support the heterogenous nature of social support perceived by bullying victims and offer insights into more tailored interventions aimed at promoting the development victims in different subgroups.

## Introduction

Many studies have shown that higher social support associates with better outcomes in bullying victims (e.g., Yeung & Leadbeater, 2013). The existing literature on school bullying and social support mainly adopts variable-centered approaches to examine the independent effects of individual sources of social support (e.g., Desjardins & Leadbeater, 2011), but adolescents’ development is shaped by the joint effects of multiple sources of social support (Bronfenbrenner, 1979). Despite extensive research, it remains unclear how multiple sources of social support aggregate at an individual level and how multiple support jointly shapes victims’ outcomes from a holistic perspective. The current study aims to contribute to the literature by using a person-centered approach to identify bullying victims’ perceived social support profiles from five sources (i.e., parents, teachers, classmates, close friends, and online-only friends) and explore how the social support profiles correspond with bullying victims’ demographic characteristics (i.e., age, gender, socioeconomic status, and family structure), frequency of victimization, psychological health (i.e., subjective well-being, depression, and anxiety), and prosocial behavior.

### School Bullying and Bullying Victims

School bullying is a form of aggressive act that can take different forms, such as physical (e.g., hitting), verbal (e.g., name-calling), indirect (e.g., excluding someone from social circles), and cyberbullying (e.g., spreading rumors online; Wang et al., 2009). School bullying could take place in any age group, but it is most evident among middle school adolescents (e.g., Zhou & Luo, 2017). Furthermore, bullying victimization can be detrimental and long-lasting, affecting many aspects of victimized adolescents’ development (McDougall & Vaillancourt, 2015). Notably, research has found that even infrequent school bullying can have adverse impacts on victims (e.g., Gower & Borowsky, 2013). As such, the assessment of bullying victimization should take into account the frequency and the different types of school bullying.

### Perceived Social Support of Bullying Victims

In line with the positive psychology movement, a wave of research has been undertaken on protective factors for bullying victims, such as social support (e.g., Yang et al., 2023). Social support refers to “an individual’s perceptions of general support or specific supportive behaviors (available or enacted on) from people in their social network, which enhances their functioning or may buffer them from adverse outcomes” (Demaray & Malecki 2002, p. 215). According to research on help-seeking behaviors of bullying victims, their primary sources of support are their parents, teachers, and peers (Humphrey & Symes, 2010). It is further demonstrated that high social support from these sources is associated with positive outcomes in bullying victims, such as high subjective well-being (e.g., Hellfeldt et al., 2020) and reduced risks of depression and anxiety (e.g., Guo et al., 2020), whereas insufficient social support consistently related to psychological health issues and problem behaviors (e.g., Holfeld & Baitz, 2020; Kong, & Lu, 2023).

While substantial evidence suggests that higher social support contributes to better outcomes in victims, some studies found opposite results (e.g., Desjardins & Leadbeater, 2011). For example, higher peer support could be linked to increased symptoms of depression in bullying victims (e.g., Desjardins & Leadbeater, 2011) due to co-rumination, where victims repeatedly recall miserable memories with their peers and dwell on negative emotions (Rose, 2021). Consequently, co-rumination could result in greater internalizing symptoms (Rose, 2021). This study viewed peers as an entire group despite the fact that peers comprise a wide range of people, from close friends who form strong social bonds to age-mates who barely know each other (Lyell et al., 2020). Therefore, it is unclear whether all peers play similar roles in victims’ outcomes or different peer groups have distinct effects. To obtain a more nuanced understanding of peer support, this study examined peer support by two specific sources: classmates and close friends.

In addition to the conventional sources of support reviewed above, the popularity of social media among young people has given rise to a new source of support- online support (Boyd & Ellison, 2007). Online support entails support offered by in-person friends (friendships that extends from offline to the online context) and online-only friends (friendships that take place exclusively online without in-person interaction; Nesi et al., 2018a). This study focused on online support provided by online-only friends to differentiate the sources of support investigated. To date, online support research has largely been undertaken with the general population and yielded divergent results. For example, support from online-only friends could have a protective effect on adolescents with suicidal ideation (e.g., Mass-Schaffer et al., 2022). In contrast, higher support from online-only friends could be associated with greater loneliness among college students (e.g., Lin, 2016). Concerning bullying victims, fewer studies have been undertaken to examine their experience with online support. One of these preliminary efforts is a survey study on online support among victims of offline bullying (Takano & Yokotani, 2022). The study showed that higher online support was related to better global mental health status and fewer symptoms of depression among victims. Given the popularity and potentially powerful role of social media among adolescents, this study extends the investigation of social support from offline sources to social support from online-only friends.

### Joint Effects of Multiple Sources of Social Support: A Person-centered Approach

According to the Bioecological System Model of Human Development, an individual’s development is shaped by the interactions between that person and their contexts and the interactions among various contexts (Bronfenbrenner, 1979). Research examining school bullying and social support has mostly centered on the *microsystem* level, the most proximal contexts to an individual (Bronfenbrenner, 1979), and it has found that social support contributes to positive development in bullying victims (e.g., Guo et al., 2020). These findings pertain to the independent effect of a particular source of social support (e.g., parent support *or* teacher support; Hellfeldt et al., 2020). From a broader perspective, adolescents interact with various microsystems simultaneously, which takes place at the *mesosystem* level (“a system of microsystems”; Bronfenbrenner, 1979, p. 25). For example, adolescents whose parents work closely with their teachers may perceive support from both their parents and teachers, and thus may have a higher chance of overcoming the negative impacts of bullying victimization. These adolescents’ development is related to the joint effects of parent support *and* teacher support, not parent support *or* teacher support. The joint effects among multiple support suggest the need to adopt a holistic view to explore various sources of social support concurrently. However, scarce studies investigate the joint effects of multiple sources of social support on bullying victims, compared to the large body of studies looking at the independent effect of a specific social support.

Methodologically, prior studies have relied on variable-centered approaches to investigate the predictors and outcomes of bullying victims’ social support (e.g., Yeung & Leadbeater, 2013). Specifically, studies using variable-centered approaches (e.g., regression) assume that their samples of bullying victims are homogeneous in terms of how social support operates on them (Laursen & Hoff, 2006). As a result, these studies shed light on the associations between social support and victims’ outcomes on average for the entire sample (Laursen & Hoff, 2006). However, not all adolescents are supported by the same pattern of support, indicating that adolescents are a heterogenous group in terms of perceived social support. Person-centered approaches, basing on population heterogeneity (Laursen & Hoff, 2006), are useful in identifying subgroups of bullying victims with similar patterns of social support and provide insights into more targeted interventions for each subgroup of victims. Despite a lack of research using person-centered approaches to explore social support among bullying victims, studies with general adolescents suggested that distinct patterns of social support operate across subgroups of adolescents. For example, a study on perceived social support of two groups of Finnish students identified four profiles (*high support*, *average support*, *low teacher support*, and *low support*) among fourth graders and three profiles (*high support*, *low support*, and *low teacher support*) among seventh graders (Ulmanen et al., 2022). Another example is a longitudinal study that identified six profiles of perceived social support (*isolated*, *weakly supported*, *fully integrated*, *parent-peer supported*, *moderately supported*, and *peer supported*) among Australian adolescents (Ciarrochi et al., 2017). Through a holistic lens, the current study investigated how five sources of social support aggregate at the level of individual bullying victims using a person-centered approach.

### Bullying Victims’ Demographic Characteristics, Frequency of Victimization, and Their Perceived Social Support

Research has established the associations between bullying victim’s demographic characteristics and their perceived social support. Studies have shown that younger kids tend to report perceiving higher support than their older counterparts (e.g., Singstad et al., 2021). Moreover, girls are consistently found to report higher overall support than boys because they are more likely to use social support as a coping mechanism and seek support from various sources (e.g., Rueger et al., 2016). Research further suggests that victims from higher-socioeconomic status (SES) families or intact families (living with both of their biological parents) are more likely to report higher social support (e.g., Schafer & Vargas, 2016). In addition to demographic characteristics, the frequency of victimization has been shown to relate to adolescents’ perceived social support. Specifically, research suggests that more frequent victimization experience is related to fewer social support, possibly due to the lower standing of victims in their social networks (e.g., Turanovic et al., 2023). Therefore, the present study examines the extent to which bullying victim’s demographic characteristics (i.e., age, gender, SES, and family structure) and frequency of victimization are associated with their social support profiles.

### Perceived Social Support and Psychological Health and Prosocial Behavior

Positive psychology looks into what and how personal strengths and environmental assets can mitigate or prevent the development of psychological issues and promote optimal development (Allen et al., 2022). Research in the field of positive psychology further suggests that a supportive interpersonal network may alleviate the adverse effects of stressful life events (e.g., school bullying) on one’s development (Jiang et al., 2019). For bullying victims, high social support from multiple sources may provide them with comfort and bolster their personal strengths (e.g., self-esteem, self-efficacy, and adaptive cognitive appraisal) to cope with the negative impacts of school bullying, which reduces the likelihood of them developing severe psychosocial issues (e.g., Guo et al., 2020).

Psychological health refers to adolescents’ cognitive and emotional appraisals of their lives, encompassing positive (e.g., subjective well-being) and negative aspects (e.g., depression and anxiety; Zhu et al., 2020). Research suggests that bullying victims with lower social support tend to report lower subjective well-being and more psychological health issues, such as severer symptoms of depression (e.g., Holfeld & Baitz, 2020), whereas those with higher social support are more likely to thrive in the aftermath of school bullying (e.g., Guo et al., 2020).

Prosocial behavior has also been extensively examined in the literature on school bullying and refers to altruistic acts intended to benefit other people, such as helping and sharing (e.g., García-Vázquez et al., 2020). Bullying victims tend to be less prosocial when there is insufficient social support (Kong, & Lu, 2023). According to the General Strain theory, school bullying can give rise to strain. Victims who lack of support may cope with their strain using maladaptive strategies, such as bullying others (Agnew, 2007). Thus, these adolescents tend to be less prosocial. Taken together, psychological health and prosocial behavior may serve as distal outcomes of the social support profiles of bullying victims.

## Current Study

Bullying victims perceive different patterns of social support and their outcomes are shaped by the joint effects of multiple sources of support. Yet few studies undertake person-centered approaches to investigate how multiple sources of social support aggregate at an individual level and how patterns of social support differ in their associations with bullying victims’ outcomes. Using a person-centered approach, this study investigates the profiles of bullying victims’ social support and explores how bullying victims’ social support profiles correspond with their psychological health and prosocial behavior. In addition, as age, gender, SES, family structure, and frequency of victimization have been found to be closely associated with the levels of social support that adolescents receive, the current study assesses their associations with bullying victims’ social support profiles. Give that this study is exploratory in nature, hypotheses that are informed by prior research were not proposed; instead, the study investigated the following research questions: Using a person-centered approach, how many profiles of social support can be identified among bullying victims and what are the features of each profile (Research Question 1)? How does each social support profile differ in their associations with bullying victims’ demographic characteristics (i.e., age, gender, SES, and family structure) and frequency of victimization (Research Question 2)? How does each social support profile differ in their associations with bullying victims’ psychological health (i.e., subjective well-being, depression, and anxiety) and prosocial behavior (Research Question 3)?

## Method

### Procedure and Participants

This study was part of a larger research project on school bullying among adolescents. Participants from nine public middle schools were recruited by convenient sampling. All of the schools are located in China, with five in Gansu Province, two in Guangdong Province, and the remaining two in Shandong Province. These schools are located in urban areas and recruit students from the neighborhood without exams or interviews. Prior to data collection, adolescents, their guardians, and schools gave assent/consent to take part in the study. Participation was voluntary and participants had the right to withdraw from the study at any time. Participants completed paper-and-pencil questionnaires at school during a 30-min session in the presence of psychology teachers. To protect participants’ privacy, identification codes were used instead of their real names. After completion, participants were debriefed and received incentives in the form of stationery. Data were collected from June to July in 2023. The study was approved by the authors’ institutional review board.

Initially, 3123 adolescents took part in the study. Among these adolescents, 581 (18.0%) did not pass validity check (i.e., failing to answer at least two out of three validity check items correctly (accuracy < 50%; Dvorsky et al., 2019)) and were excluded. Of the remaining 2542 adolescents, 36 (1.4%) did not live with at least one parent and 1607 (63.2%) did not report being bullied at least once in the current semester. As such, a total of 915 (51.9% boys; age: *M* = 13.52, *SD* = 0.75) participants were retained for the present study. Most of the participants were in Grade 7 (54.3%) or Grade 8 (44.8%), and only nine (0.9%) were in Grade 9^1^. A majority of the participants came from families in which both parents were married (84.5%), the remaining came from single-parent (8.2%) or blended families (6.7%), or did not report their family structure (0.7%). In terms of school bullying, 30.9, 80.5, 62.8, and 13.2% of the participants reported being physically, verbally, indirectly, or cyber bullied, respectively, at least once in the current semester.

### Measures

#### Victimization Experience

Definitions of school bullying were included at the beginning of the questionnaire to facilitate participants’ understanding of bullying behaviors. Six items of the Chinese version of the revised Olweus Bully/Victim Questionnaire (Li et al., 2012) and one item of the Chinese version of the Cyberbullying Survey (Li, 2007) were used to assess physical, verbal, indirect, and cyber bullying. A sample item is “I was hit, kicked, pushed, beaten, or threatened by my classmate(s).” Participants were asked to report their victimization experience in the current semester using a 5-point Likert scale (1 = *Not at all*, 2 = *Once or twice in the current semester*, 3 = *Two or three times a month*, 4 = *About once a week*, 5 = *Several times a week*). Higher scores indicate more frequent bullying victimization. The Cronbach *α* value of the seven victimization items was 0.78 in the present study. Empirical evidence has demonstrated that even infrequent victimization experience can have negative impacts on adolescents’ psychological and social outcomes (Gower & Borowsky, 2013). Therefore, adolescents who reported experiencing victimization at least once in the past four months on any of the six victimization items were included in this study.

#### Perceived Social Support

Perceived social support from parents, teachers, classmates, and close friends was assessed by the Chinese version of the Child and Adolescent Social Support Scale (CASSS; Zhao et al., 2021; Malecki et al. 2003). Perceived support from online-only friends was examined by adapting the close friend subscale of the CASSS. Specifically, “close friend” was replaced by “online-only friend”. Adolescents rated a total of 60 items (i.e., 12 items for each source of support) on a 6-point Likert scale from 1 = *Never* to 6 = *Always*. Sample items are “My parents listen to me when I need to talk (parent support)”, “My teachers care about me (teacher support)”, “My classmates give me good advice (classmate support)”, My close friend understands my feelings (close friend support), and “My online-only friend understands my feelings (online-only friend support)”. Items measuring the same source of social support were summed to create a social support score for each source, with a higher score indicating a higher level of support from that particular source. The measure had been validated on Chinese adolescents (Li et al., 2021) and exhibited excellent internal reliability in this study (*α* = 0.94 for parent support, *α* = 0.94 for teacher support, *α* = 0.95 for classmate support, *α* = 0.96 for close friend support; and *α* = 0.98 for online support). Additionally, the confirmatory factor analysis supported the validity of the online support subscale (comparative fit index: 0.980; Tucker-Lewis index: 0.972; root mean square error of approximation: 0.068; Boateng et al., 2018).

#### Subjective Well-Being

Subjective well-being was assessed in two parts: life satisfaction and positive and negative affect (Diener et al., 2017). Life satisfaction was assessed by the Chinese version of the Brief Multidimensional Students’ Life Satisfaction Scale (BMSLSS; Jiang et al., 2018). The BMSLSS measures school-aged children’s (ages 8–18) satisfaction in five specific domains (i.e., school, family, friends, self, and living environment) with one item tapping into each domain. Adolescents rated their satisfaction on a 7-point Likert scale from 1 = *Very unsatisfied* to 7 = *Very satisfied*. The sum of the five life satisfaction items was calculated, with higher numbers indicating higher levels of life satisfaction. An additional item was included to assess adolescents’ global life satisfaction and serve as a validity check for the BMSLSS. Score for the additional item was highly correlated with the BMSLSS total score in this study (*r* =0.82, *p* < 0.001), suggesting that the BMSLSS could be used with confidence in the analyses (Tian et al., 2015). The Chinese BMSLSS has been validated in the Chinese adolescent population (*α* = 0.77; Jiang et al., 2018). The Cronbach *α* value was 0.83 in this study.

Positive affect (PA) and negative affect (NA) were assessed by the Chinese version of the Positive and Negative Affect Schedule (Huang et al., 2003; Watson et al., 1988). Adolescents indicated how they felt in the current semester by responding to ten PA items (e.g., interested, excited, enthusiastic) and ten NA items (e.g., distress, upset, guilty) on a 5-point Likert scale, ranging from 1 = *Very slightly or not at all* to 5 = *Extremely*. The sum of the items measuring each affect was taken, with higher numbers indicating greater affect. The Cronbach *α* values were 0.84 and 0.85 for PA and NA, respectively, in the present study. To create the subjective well-being score, participants’ BMSLSS total score was added to their PA score minus their NA score (Tian et al., 2016).

#### Depression

Symptoms of depression were examined by the Chinese version of the 9-item Patient Health Questionnaire (PHQ-9; Kroenke et al., 2001; Yang et al., 2015). Adolescents reported how often they felt a particular way in the past two weeks, using a 4-point Likert scale (0 = *Not at all* to 3 = *Nearly every day*). A sample item is “Little interest or pleasure in doing things.” Items were summed for a total score, with higher scores indicate greater symptoms of depression. The Chinese PHQ-9 has been validated in the Chinese adolescent population (*α* = 0.91; Peng et al., 2022) and exhibited good internal reliability (*α* = 0.86) in the present study.

#### Anxiety

Symptoms of anxiety were assessed by the Chinese version of the 7-item Generalized Anxiety Disorder Scale (GAD-7; He et al., 2010; Spitzer et al., 2006). Adolescents reported the frequency of seven symptoms of anxiety during the past two weeks by responding to the items on a 4-point Likert scale (0 = *Not at all* to 3 = *Nearly every day*). A sample item is “Feeling afraid as if something awful might happen.” Items were summed for a total score, with higher total scores indicate greater symptoms of anxiety. The Chinese GAD-7 has been validated in the Chinese adolescent population (*α* = 0.93; Peng et al., 2022) and exhibited excellent internal reliability (*α* = 0.90) in the present study.

#### Prosocial Behavior

Prosocial behavior was assessed by the prosocial behavior subscale of the Chinese version of the Strengths and Difficulties Questionnaire (SDQ; Goodman, 2001). Five items were rated on a 3-point Likert scale (i.e., 0 = *Not true*, 1 = *Somewhat true*, 2 = *Certainly true*). A sample item is “I am helpful if someone is hurt, upset or feeling ill”. The Cronbach *α* value was 0.73 in the present study.

#### Demographic Characteristics

Adolescents were asked about their age, gender, living condition, family structure, family income, and parental education and occupation. To calculate an SES score, family income^2^ (1 = *5000 RMB or below* to 5 = *More than 30,000 RMB*; 1 RMB = 0.14 USD), parental education (1 = *Never attended school* to 6 = *Graduate degree or above*), and occupational prestige (1 = *Peasant or jobless*, 2 = *Blue collar*, and 3 = *Professional or semi-professional occupations*) were standardized and averaged to index the family SES (Luo et al., 2023; Song et al., 2023), with a higher number indicating higher SES.

### Analytic Plan

Preliminary analyses were conducted using SPSS 26 to generate descriptive statistics. To identify bullying victims’ social support profiles, latent profile analysis (LPA) was conducted using M*Plus* 8.3 (Muthén & Muthén, 1998–2017). The five sources of social support were entered as indicators. LPA solutions were generated, ranging from one to eight profiles, using maximum likelihood estimation. The following indices of fit were consulted to ascertain the adequacy of solutions: Akaike Information Criterion (AIC), consistent Akaike information criterion (CAIC), Bayesian information criterion (BIC), sample-size adjusted Bayesian information criterion (SSA-BIC), Lo-Mendell-Rubins likelihood ratio test (LMR), bootstrap likelihood ratio test, average latent class posterior probabilities, and entropy (Morin et al., 2016; Tein et al., 2013). As suggested, lower AIC, CAIC, BIC, and SSA-BIC values reflect better profile solutions (Nylund et al., 2007). The LMR and bootstrap likelihood ratio test compare between a *k* profile solution and a *k*-1 profile solution. A significant *p*-value suggests that the *k* profile solution fits better than *k*-1 profile solution. Average latent profile posterior probabilities reveal whether participants are accurately classified into their most likely profile, with values above 70% being satisfactory (Nylund et al., 2007). Entropy values reflect the accuracy of classification, with higher values indicating more precise classification. Once the optimal profile solution was determined, the R3STEP command was employed to examine the associations between adolescents’ demographic characteristics, frequency of victimization, and social support profiles (Asparouhov & Muthén, 2014a, 2014b). If the 95% confidence interval of the odds ratio does not include one, the results are said to be significant. Lastly, associations between each social support profile and psychological health and prosocial behavior were assessed using the BCH method (Asparouhov & Muthén, 2014a, 2014b). The BCH method examined mean-level differences in the distal outcomes across social support profiles while retaining the composition of the identified profiles.

## Results

### Preliminary Analyses

Missing data at the item level accounted for 0.0–2.5% of all included participants’ responses. Missing Completely At Random test (Little & Rubin, 1989) showed a normed chi-square (*χ*^2^/*df*) of 1.39, suggesting a random missing pattern (Bollen, 1989). Hence, full information maximum likelihood was used to handle missing data. Table 1 presents descriptive statistics of study variables. Except for SES, all variables exhibited values of skewness and kurtosis ranging between −2 and +2 (George & Mallery, 2010), suggesting normal distributions.

**Table 1:** Descriptive Statistics of All Variables (*N* = 915)

Variables	*M*	*SD*	Skewness	Kurtosis
Profile indicators				
Parent support	39.88	13.97	0.31	−0.63
Teacher support	52.26	13.04	−0.36	−0.44
Classmate support	46.71	14.20	−0.14	−0.63
Close friend support	49.29	14.77	−0.25	−0.64
Online support	33.24	18.94	0.39	−1.03
Demographic characteristics				
Age	13.52	0.75	0.38	0.18
Gender^a^	0.52	0.50	−0.08	−1.99
SES	−0.09	0.51	1.11	4.11
Distal outcomes				
SWB^b^	28.93	15.16	−0.14	−0.17
Depression	9.93	6.01	0.54	−0.18
Anxiety	7.66	5.49	0.58	−0.46
Prosocial behavior	7.45	2.11	−0.61	−0.23

^a^Gender was coded as: 0 = boy, 1 = girl

^b^SWB = subjective well-being and was calculated as life satisfaction + (positive affect − negative affect; Tian et al., 2016)

### LPA Profile Composition

Table 2 demonstrates the fit statistics for the LPA solutions with one through eight profiles. The AIC, CAIC, BIC, and SSA-BIC values decreased as the number of profiles increased. The six- to eight-profile solutions were excluded as their LMP and BLRT *p-*values were not significant. Among the remaining five solutions, results suggested the five-profile solution to be optimal. Specifically, although the five-profile solution did not show the highest entropy value, it yielded significant LMR and BLRT results, suggesting that the five-profile solution was superior to the four-profile solution. Moreover, the five-profile solution demonstrated satisfactory results for average latent profile posterior probabilities, ranging from 85 to 88%, suggesting accurate profile classification.

**Table 2:** Model Fit Indices for the Latent Profile Analysis

Number of profiles	Parameter estimates	AIC CAIC	BIC	SSA-BIC	Entropy	LMR (*p*)	BLRT (*p*)	Profile proportions
1	10	37132.056 37151.670	37180.246	37148.487				
2	16	36364.493 36395.877	36441.596	36390.782	0.758	<0.001	<0.001	47.5, 52.5
3	22	36189.505 36232.657	36295.521	36225.652	0.765	<0.01	<0.01	15.6, 36.6, 47.8
4	28	36123.028 36177.948	36257.958	36169.034	0.837	<0.01	<0.01	3.3, 14.3, 37.4, 45.0
**5**	**34**	**36068.417 36135.106**	**36232.260**	**36124.281**	**0.798**	**<0.05**	**<0.05**	**3.3, 14.5, 18.1, 19.0, 45.1**
6	40	35960.007 36059.107	36152.764	36025.729	0.831	0.055	0.058	7.1, 12.1, 16.5, 19.4, 21.9, 23.0
7	46	35892.817 35983.043	36114.488	35968.398	0.824	0.093	0.097	6.8, 6.9, 12.0, 15.8, 17.8, 20.3, 20.5
8	52	35844.845 35971.966	36095.429	35930.283	0.823	0.667	0.673	5.3, 6.3, 7.1, 9.7, 13.9, 16.3, 19.8, 21.5

The optimal model is shown in bold

*AIC* Akaike information criterion, *BIC* Bayesian information criterion, *SSA-BIC* sample-size adjusted BIC, *LMR* Lo, Mendell, and Rubin likelihood ratio test, *BLRT* bootstrap likelihood ratio test

**Fig. 1:**
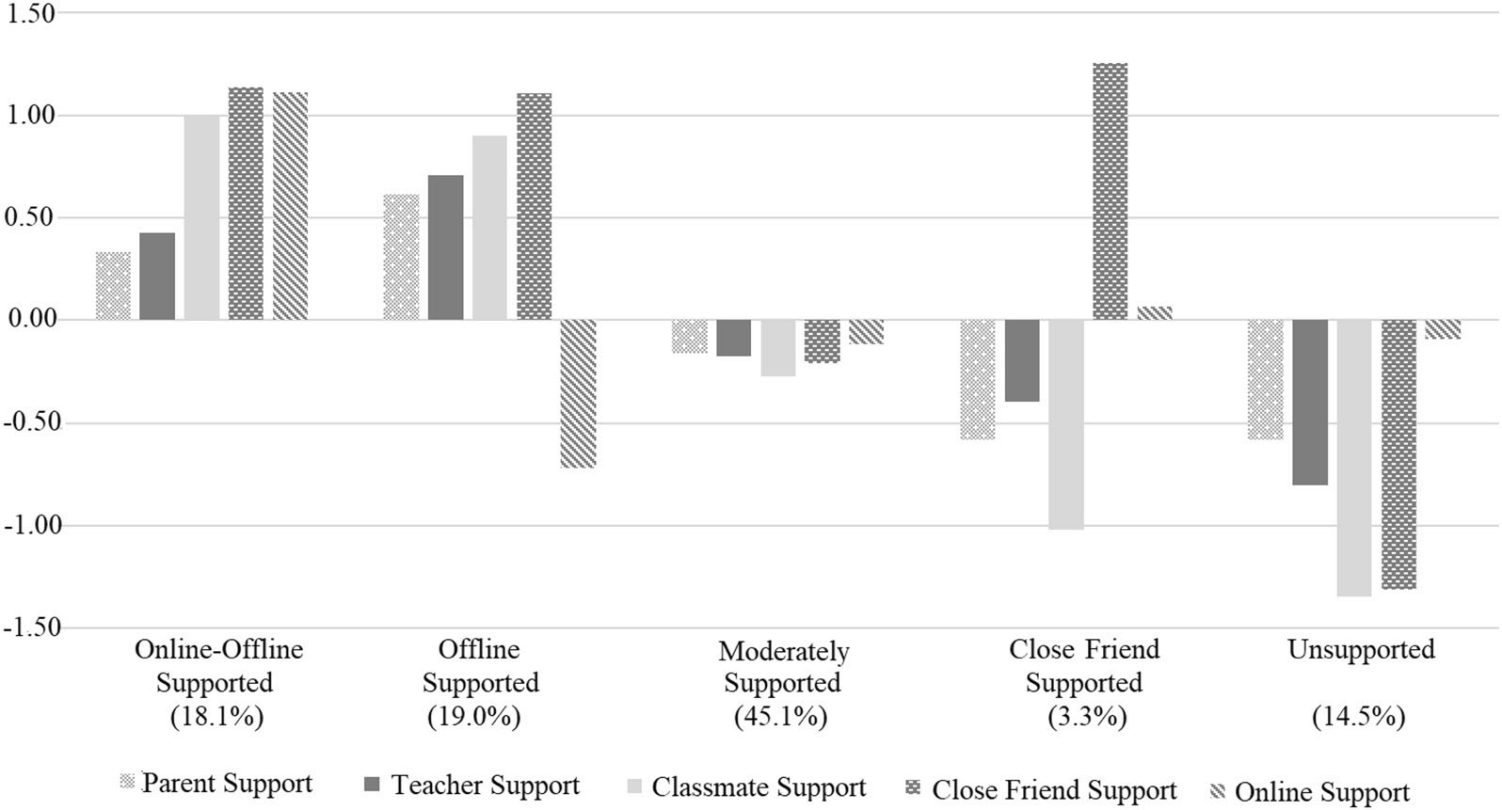
Standardized Mean Estimates of Bullying Victims’ Social Support Profiles

Profile 1 consisted of 18.1% of participants (*n* = 166) and was labelled as the *online-offline supported* profile because bullying victims belonging to this profile reported high levels of support from all five sources, both online and offline. Next, Profile 2 included 19.0% of participants (*n* = 174) and was labelled as the *offline supported* profile because victims belonging to this profile reported high levels of support from parents, teachers, close friends, and classmates (offline sources) but the lowest levels of online support. Profile 3 comprised the majority of participants (45.1% of sample, *n* = 413). These bullying victims reported average levels of support from all five sources, and thus Profile 3 were labelled as the *moderately supported* profile. Profile 4 consisted of 3.3% of participants (*n* = 30) and was labelled as the *close friend supported* profile because victims belonging to this profile reported the highest level of close friend support, along with average level of online support and low levels of support from parents, teachers, and classmates. Finally, Profile 5 included 14.5% participants (*n* = 132) and was labelled as the *unsupported* profile because victims belonging to this profile reported the lowest levels of teacher, classmate, and close friend support, low level of parent support, and average level of online support. Table 3 presents descriptive statistics (i.e., mean and standard error) of the profiles and mean differences of indicators between profiles. Figure 1 depicts the standardized estimates (i.e., z-score) of the analysis.

**Table 3:** Mean Differences of Indicators Between Social Support Profiles

Indicators	Online-Offline Supported (*n* = 166)		Offline Supported (*n* = 174)	Moderately Supported (*n* = 413)	Close friend Supported (*n* = 30)	Unsupported (*n* = 132)	*F*	Partial *η*^2^
Parent support		44.54_a_ (1.72)	48.42_a_ (1.64)	37.63_b_ (0.77)	31.75_c_ (2.67)	31.80_c_ (1.17)	44.089***	0.16
Teacher support		57.79_a_ (1.36)	61.47_a_ (1.09)	49.95_b_ (0.88)	47.04_b, c_ (6.24)	41.79_c_ (1.62)	78.664***	0.26
Classmate support		60.93_a_ (1.02)	59.49_a_ (0.97)	42.83_b_ (0.94)	32.23_c_ (4.57)	27.64_c_ (1.30)	487.757***	0.69
Close friend support		63.46_a_ (0.84)	63.09_a_ (0.80)	43.59_b_ (0.80)	65.21_a_ (1.73)	27.34_c_ (2.11)	825.426***	0.79
Online support		54.25_a_ (1.73)	19.60_c_ (1.25)	31.01_b_ (0.98)	34.51_b_ (5.40)	31.44_b_ (2.02)	147.490***	0.40

Standard deviations are presented in brackets. Means with different subscript letters were significantly differed at *p* < 0.005 (Bonferroni adjusted alpha level). Partial *η*^2^ = 0.01–0.06 indicates a small effect size, 0.06–0.14 a moderate effect size, and > 0.14 a large effect size (Cohen, 1988)

***p < 0.001

Since the *close friend supported* profile is relatively small, sensitivity analysis was conducted to estimate and confirm its robustness. Notably, this profile was also identified in the four-profile solution (Appendix A), which displayed the highest entropy (0.837) among the eight solutions. Therefore, the *close friend supported* profile did consistently appear in different models, indicating that it reflects a modest yet well-defined group of bullying victims.

### Associations between Bullying Victims’ Demographic Characteristics, Frequency of Victimization, and Social Support Profiles

The associations between bullying victims’ demographic characteristics (i.e., age, gender, SES, and family structure), frequency of victimization, and social support profiles were investigated (see Table 4). In terms of age, younger adolescents were more likely to be in the *offline supported* profile than the *online-offline supported*, *moderately supported* and *unsupported* profiles. Gender differences were also evident. Girls were more likely to be in the *online-offline supported* profile, when compared with the *moderately supported* profile. Additionally, bullied adolescents who came from higher-SES families were more likely to be in the *offline supported* profile than the *online-offline supported*, *moderately supported* and *close friend supported* profiles. The five profiles did not differ significantly in terms of family structure. As for frequency of victimization, victims who reported more frequent victimization experience were more likely to be in the *close friend supported* and *unsupported* profiles than the other three profiles. Furthermore, victims who reported experiencing victimization more often were more likely to be in the *moderately supported* profile than the *offline supported* profile.

**Table 4:** Associations between Bullying Victims’ Demographic Characteristics, Frequency of Victimization, and Social Support Profiles

	*OR*	95% CI	*OR*	95% CI	*OR*	95% CI	*OR*	95% CI	*OR*	95% CI
Variables	Online-Offline Supported vs Offline Supported (Re)		Online-Offline Supported vs Moderately Supported (Re)		Online-Offline Supported vs Close friend Supported (Re)		Online-Offline Supported vs Unsupported (Re)		Offline Supported vs Moderately Supported (Re)	
Age	**1.62**	**[1.085, 2.412]**	1.04	[0.780, 1.385]	1.05	[0.317, 3.495]	0.81	[0.548, 1.200]	**0.64**	**[0.460, 0.897]**
Gender^a^	1.63	[0.889, 2.971]	**1.66**	**[1.055, 2.597]**	1.29	[0.419, 3.988]	1.14	[0.619, 2.093]	1.02	[0.639, 1.624]
SES	**0.46**	**[0.267, 0.799]**	0.92	[0.578, 1.471]	1.05	[0.488, 2.265]	0.75	[0.399, 1.394]	**2.00**	**[1.287, 3.100]**
Family Structure	0.82	[0.436, 1.540]	0.90	[0.577, 1.402]	1.00	[0.324, 3.083]	0.75	[0.443, 1.266]	1.10	[0.678, 1.776]
Frequency of Victimization	1.18	[0.965, 1.430]	0.90	[0.805, 1.007]	**0.76**	**[0.657, 0.889]**	**0.78**	**[0.696, 0.880]**	**0.77**	**[0.659, 0.891]**

Variables	Offline Supported vs Close friend Supported (Re)		Offline Supported vs Unsupported (Re)		Moderately Supported vs Close friend Supported (Re)		Moderately Supported vs Unsupported (Re)		Close friend Supported vs Unsupported (Re)	
Age	0.65	[0.198, 2.142]	**0.50**	**[0.327, 0.768]**	1.01	[0.320, 3.210]	0.78	[0.551, 1.106]	0.77	[0.254, 2.336]
Gender^a^	0.80	[0.256, 2.474]	0.70	[0.375, 1.308]	0.78	[0.266, 2.289]	0.69	[0.398, 1.187]	0.88	[0.302, 2.566]
SES	**2.28**	**[1.085, 4.780]**	1.62	[0.883, 2.956]	1.14	[0.573, 2.270]	0.81	[0.453, 1.443]	0.71	[0.330, 1.524]
Family Structure	1.22	[0.385, 3.868]	0.91	[0.530, 1.577]	1.11	[0.385, 3.208]	0.83	[0.559, 1.241]	0.75	[0.262, 2.140]
Frequency of Victimization	**0.65**	**[0.544, 0.777]**	**0.67**	**[0.570, 0.778]**	**0.85**	**[0.773, 0.932]**	**0.87**	**[0.829, 0.911]**	1.02	[0.939, 1.116]

Significant results are indicated in bold

^a^Gender was coded as: 0 = boy, 1 = girl

*SES* family socioeconomic status, *Re* reference group, *OR* odds ratio, 95% CI = 95% confidence interval

### Associations between Social Support Profiles and Psychological Health and Prosocial Behavior

Table 5 shows the associations between bullying victims’ social support profiles and psychological health and prosocial behavior. Significant chi-squared statistics were observed for all distal outcomes. Specifically, in terms of psychological health, *offline supported* adolescents reported the highest subjective well-being, followed by *online-offline supported*, *moderately supported* adolescents, and lowest among *unsupported* and *close friend supported* adolescents. *Offline supported* adolescents also reported the fewest symptoms of depression and anxiety, followed by *online-offline supported* and *moderately supported* adolescents, and *unsupported* and *close friend supported* adolescents reported the highest number of symptoms of depression and anxiety. As for prosocial behavior, *online-offline supported* and *offline supported* adolescents reported more prosocial behaviors than *moderately supported*, *close friend supported*, and *unsupported* adolescents.

**Table 5:** Associations between Social Support Profiles and Psychological Health and Prosocial Behavior

Outcomes	Online-Offline Supported	Offline Supported	Moderately Supported	Close friend Supported	Unsupported	*χ*^2^	Cramer’s *V*
SWB	34.83_b_ (1.25)	40.79_a_ (1.30)	27.30_c_ (0.73)	13.98_d_ (3.53)	14.04_d_ (1.33)	282.398***	0.32
Depression	9.63_b_ (0.52)	6.25_c_ (0.50)	9.82_b_ (0.31)	16.66_a_ (1.48)	13.89_a_ (0.67)	111.473***	0.20
Anxiety	7.27_b_ (0.48)	5.15_c_ (0.45)	7.31_b_ (0.29)	12.25_a_ (1.51)	11.49_a_ (0.60)	83.507***	0.17
Prosocial behavior	8.60_a_ (0.16)	8.31_a_ (0.18)	7.08_b_ (0.11)	6.59_b_ (0.48)	6.27_b_ (0.24)	133.253***	0.22

Means with different subscript letters were significantly differed at *p* < 0.005 (Bonferroni adjusted alpha level). Based on the degree of freedom in the model, Cramer’s *V* = 0.06 indicates a small effect size, 0.17 a moderate effect size, and 0.29 a large effect size (Cohen, 1988)

***p < 0.001

SWB subjective well-being

## Discussion

Prior research on bullying victims’ perceived social support have mostly used variable-centered approaches, overlooking the existence of distinct patterns of social support or possible differences in the associations between social support patterns and bullying victims’ outcomes. The present study explored the profiles of bullying victims’ perceived social support and investigated how these profiles differ in their associations with bullied adolescents’ demographic characteristics, psychological health, and prosocial behavior. LPA identified five profiles: *online-offline supported* adolescents, *offline supported* adolescents, *moderately supported* adolescents, *close friend supported* adolescents, and *unsupported* adolescents. Furthermore, results indicate that the five profiles correspond differently to bullying victims’ demographic characteristics (i.e., age, gender, and SES), frequency of victimization, psychological health (i.e., subjective well-being, depression, and anxiety), and prosocial behavior.

### Bullying Victims’ Profiles of Perceived Social Support

Our study identified five profiles of perceived social support among bullied adolescents. *Online-offline supported* adolescents were found to perceive the highest social support from all sources. *Offline supported* adolescents reported high levels of support from offline resources (i.e., parents, teachers, classmates, and close friends) but the lowest levels of support from online-only friends. Bullying victims belonging to these two profiles are said to perceive high social support. Next, *moderately supported* adolescents reported average levels of support from all five sources and made up the largest group, accounting for roughly 45% of the sample. This finding implies that about half of the bullying victims got social support, to some extent. C*lose friend supported* adolescents reported average to low levels of support from their parents, teachers, classmates, and online-only friends but the highest level of support from close friends, indicating that their close friends are their main source of social support. This profile reflects the developmental trajectory in that adolescents tend to shift their primary social support network away from parents to peers of their own age (Rueger et al., 2016). Lastly, *unsupported* adolescents reported the lowest levels of teacher, classmate, and close friend support, low level of parent support, but average level of online support. While bullying victims falling into this profile lack support from offline resources, they nonetheless perceive some online support. Taken together, the five identified profiles demonstrate different combinations of levels of social support from multiple sources, indicating that bullying victims are indeed a heterogeneous group in terms of their social support patterns. Moreover, none of the profiles manifest low support from all sources, suggesting that all bullying victims in this study perceive some sort of support from at least one source.

The highly (i.e., *online-offline supported* and *offline supported*), *moderately*, and *unsupported* profiles identified in the study with bullying victims replicate the *high support*, *moderate support*, *low support* profiles identified in studies with general adolescents (e.g., Chan et al., 2022), suggesting that these profiles are consistently present among adolescents with or without bullying victimization. More importantly, by incorporating support from online-only friends in the assessment, the present study was able to differentiate between *online-offline supported* and *offline supported* victims among the two highly supported profiles. Furthermore, unlike many studies on social support that tend to regard peers as an entire group (e.g., Chan et al., 2022), this study assessed peer support by more specific sources (i.e., classmates, close friends). This is because peers represent a diverse group that can consist of people ranging from close friends to age-mates who may not know one another well (Lyell et al., 2020). Indeed, the findings support the idea that peers constitute a divergent group in that classmate support tends to go hand in hand with parent and teacher support (i.e., all low, all moderate, all high), whereas close friend support can occasionally stand out in the context of low overall support (i.e., *close friend supported*). Taken together, the findings allow for a more sophisticated understanding of bullying victim’ perceived social support network, which could inform the design of more targeted interventions to meet the specific needs of victims from each profile.

### Bullying Victims’ Demographic Characteristics, Frequency of Victimization, and Their Social Support Profiles

Our results reveal clear associations between demographic characteristics and bullying victims’ social support profiles. Specifically, younger adolescents are more likely to fall into the *offline supported* profile than the *online-offline supported*, *moderately supported* and *unsupported* profiles. Given that younger adolescents are less independent and require greater support, it is intuitive that people may pay more attention, care, and support to them (e.g., Singstad et al., 2021). Older adolescents, on the other hand, tend to handle school bullying on their own because of a desire for independence and control in life (Shaffer & Kipp, 2014). In terms of gender, girls were more likely to be classified as *online-offline supported*, whereas boys were more likely to be categorised as *moderately supported*. This finding corroborates many studies that found girls reported perceiving greater support than boys (e.g., Rueger et al., 2016) and aligns with the literature on coping strategy, which shows that girls are more likely than boys to use social support as a coping strategy and seek support from various sources (e.g., Rueger et al., 2010). Finally, adolescents from higher-SES families were more likely to fall into the *offline supported* profile than the *online-offline supported*, *moderately supported*, and *close friend supported* profiles. According to the Family Investment Model, higher-SES families are more resourceful and willing to support their children than lower-SES families (Conger & Donnellan, 2007). With these resources and support, higher-SES adolescents tend to perform better academically, enjoy higher social standing, establish stronger relationships with teachers and classmates, and as a result, get more social support from teachers and classmates as well (Chiu & Chow, 2015; Schafer & Vargas, 2016).

In terms of the associations between frequency of victimization and profiles of perceived social support, results suggest that victims who reported experiencing victimization more often were more likely to be in the *close friend supported* and *unsupported* profiles than the *offline supported*, *online-offline supported*, and *moderately supported* profiles. Compared to the *offline supported* profile, victims who reported more frequent victimization experience were more likely to be in the *moderately supported* profile. These results indicate that adolescents who are bullied more often tend to perceive lower levels and fewer sources of support from their social network. This could pertain to the low social standing typically associated with bullying victims. For example, adolescents place a great importance to peer status, so they often avoid making friends with their bullied peers (Turanovic et al., 2023). As a result, bullying victims generally receive low overall social support. Alternatively, it could be the other way around, with adolescents who lack social support being more likely to be bullied (Lee et al., 2022). Since the direction of relationship between frequency of victimization and social support cannot be determined in cross-sectional studies, future research may benefit from a longitudinal study to untangle this temporal relationship.

### Bullying Victims’ Social Support Profiles and Their Psychological Health and Prosocial Behavior

While both *offline-supported* and *online-offline supported* profiles of bullied adolescents perceived receiving high social support from multiple sources, *offline-supported* adolescents report higher subjective well-being and fewer symptoms of depression and anxiety than *online-offline supported* adolescents (they reported similar levels of prosocial behaviors). These results echo Longest and Kang’s (2022) study, which found that people have better psychological health when they receive high offline support accompanied with low online support. When bullied adolescents seek support from online communities, they may get support but they may also be exposed to unsupportive or even damaging information (Longest & Kang, 2022). To receive more online support, these adolescents may spend more time online, which could contribute to the development of social media addiction and ultimately exacerbate their psychological issues (Han et al., 2019). Nevertheless, these two profiles of bullying victims reported better psychological health and more prosocial behavior than victims in the other three profiles. This is in accordance with positive psychology, which proposes that greater support from multiple sources could shield bullying victims from the adverse effects of victimization experience, and subsequently contributing to improved outcomes for them (Jiang et al., 2019). Similar findings have been observed in prior studies that found adolescents with high overall social support perform better (e.g., Chan et al., 2022). The finding also signifies the importance of establishing a comprehensive social support network that involves various stakeholders to optimize the development of bullying victims.

*Moderately supported* adolescents reported a similar level of prosocial behavior to *close friend supported* and *unsupported* adolescents, but showed better subjective well-being and lower depression and anxiety. On the contrary, *close friend supported* and *unsupported* adolescents were substantially worse than the other three profiles, with adolescents in these two profiles reporting the lowest subjective well-being and the highest number of symptoms of depression and anxiety. Despite slight differences in the configuration of support, adolescents falling into the *close friend supported* and the *unsupported* profiles perceived low overall social support. Ample studies document that insufficient social support is associated with greater psychosocial problems (e.g., Holfeld & Baitz, 2020; Kong & Lu, 2023). Interestingly, the similar psychological and prosocial outcomes of *close friend supported* and *unsupported* adolescents suggest that high close friend support may not compensate for the lack of support from parents, teachers, and classmates. The positive association between close friend support and severity of mental illness had been documented in prior studies (e.g., Desjardins & Leadbeater, 2011) and could be attributed to co-rumination. When bullied adolescents turn to their close friends for support, they often dwell on unpleasant memories and negative emotions. These co-ruminative behaviors could magnify the harmful consequences of bullying victimization (Rose, 2021). Another possible explanation pertains to the lack of a comprehensive social support network. Theoretical principles and empirical evidence suggest that the optimal development of an individual depends on the joint effects of support from multiple sources (Bronfenbrenner, 1979; Ciarrochi et al., 2017). As such, when high close friend support is companied with other sources of support (i.e., the *offline supported* and *online-offline supported* profiles), bullying victims tend to report better psychological and prosocial outcomes. Conversely, when high close friend support is perceived as the only social support, victims’ psychological and prosocial outcomes are jeopardized.

Our study provides evidence for the Bioecological System Model. First, demographic characteristics vary in their associations with bullying victims’ social support profiles. For example, younger and higher-SES adolescents were more likely to belong to the *offline supported* than the *online-offline supported* profile, suggesting that victims’ characteristics contribute to the environment that they find themselves in. Second, bullying victims’ social support profiles correspond with different outcomes. For instance, although both *online-offline supported* and *offline supported* bullying victims reported receiving high multi-source support, their psychological and prosocial outcomes are not identical, with *offline supported* victims outperforming *online-offline supported* victims in terms of subjective well-being, depression, and anxiety. Disparities in psychological health outcomes between these two profiles of victims could be a result of the joint effects of the support that they get (e.g., the negative effects of online support cancelling out the positive effects of offline support). Collectively, these findings corroborate a central tenet of the Bioecological System Model, which states that individuals’ development is shaped by the interplay between their characteristics and their contexts, and the interplay among various contexts (e.g., family and school).

### Implications

The five profiles identified in the study provide empirical support to the concept that bullying victims are a heterogenous population, displaying various patterns of social support. Furthermore, the *close friend supported* profile aligns with the developmental characteristics of adolescence, as young people typically place a high value on friendship. The findings also stress that despite adolescents’ strong desire for independence, support from close relationships remains important for their positive development. Specifically, high and co-existing support from multiple offline sources contributes to optimal psychological health and prosociality among bullied adolescents. Yet, roughly 63% of the victims (i.e., the *moderately supported*, *close friend supported*, and *unsupported* profiles) did not get sufficient social support. In addition to school-based programs, holistic social support interventions that extend beyond the school context may be needed to strengthen the social support network of bullying victims. It is encouraged that different stakeholders, such as parents, teachers, peers, and social workers to collaborate to build a comprehensive social support network across family, school, and community contexts. Given the substantial differences in social support configurations among the five groups of bullying victims, a one-size-fits-all program is unlikely to ameliorate the adverse effects of bullying victimization on all of them. The findings also have specific implications for each subgroup of bullied adolescents.

First, *online-offline supported* adolescents performed worse than *offline supported* adolescents regarding psychological health, which could be due to inappropriate social media use. Parental involvement, such as monitoring and active mediation (e.g., parents discussing social media use with their child without interfering by setting rules) may reduce the adverse impacts of social media on victims who seek online support (Nielsen et al., 2019). Second, the *moderately supported* profile accounts for 45.1% of the sample, suggesting that nearly half of the bullied adolescents perceived some but insufficient support. A key barrier to getting more support pertains to negative help-seeking attitudes (e.g., seeking support as a sign of weakness; Dennehy et al., 2020). Help-seeking stigma reduction and help-seeking skills programs may facilitate bullying victims’ help-seeking process. This could include, for example, the Youth Aware of Mental Health intervention, a school-based mental health promotion intervention in which specific help-seeking skills are taught (Lindow et al., 2020). Third, *close friend supported* adolescents showed suboptimal results across all outcomes, which may be attributed to co-rumination between them and their close friends. Efforts to prevent co-rumination could include educating adolescents about it and teaching them alternative coping mechanisms, such as problem-focused and cognitive coping strategies (Rose, 2021). Fourth, *unsupported* adolescents perceive little or no support from any sources and may lack close ties with others. These bullying victims could benefit from programs like Group Teen Triple P (a parenting program aiming to promote parenting practices and parent-adolescent relationships; Chu et al., 2015) and Establish-Maintain-Restore (a teacher training program aiming to improve teachers’ skills in cultivating relationships with students; Duong et al., 2019). Moreover, as adolescents often turn to their school peers for support, training students to provide active support to victims may be effective (Salmivalli et al., 2021). Fifth, since bullying victims’ demographic characteristics are related to their perceived social support profiles, interventions should take into consideration their demographic characteristics. For example, older adolescents perceived less social support than their younger counterparts. Adults are encouraged to provide older bullying victims with age-appropriate support that respects their autonomy and independence while offering guidance and strategies to cope with the harmful impacts of school bullying (Radez et al., 2021; Wight et al., 2006). Lastly, given that victims who experienced school bullying more frequently were less likely to perceive high social support from multiple sources, it is recommended that future interventions closely monitor the frequency of bullying victimization, focus special attention on adolescents who are bullied often, and foster comprehensive social support networks for them.

### Limitations and Future Directions

This study advances the understanding of perceived social support profiles among bullying victims, but it has some limitations. First, the findings were based on adolescents’ self-reports. Although self-reported measures are a reliable method to assess subjective constructs (e.g., perceived social support), they could also result in response bias because participants may provide answers that are socially favorable (e.g., intentionally report more prosocial behaviors than they actually engage in; Greene, 2015). Future research may employ multiple methods (e.g., teacher reports) to triangulate the results, especially when it comes to behavioral constructs. Second, the nature of cross-sectional design prevents causal inferences. For example, seeking online support could contribute to more psychosocial problems, but psychosocial problems could also influence adolescents’ help-seeking behavior (Han et al., 2019). Future research may use a longitudinal design, where repeated observations made over time enable researchers to reveal the temporal associations between bullying victims’ social support and their outcomes. Lastly, the measures of victimization experience and psychological health indicators (i.e., depression and anxiety) are based on different time scales. For example, victimization experience was assessed over a four-month period while depression and anxiety were assessed over a two-week period. Using a consistent time scale across variables of interest may help to lessen the potential influence of confounding variables.

## Conclusion

Research on bullying victims’ perceived social support typically used variable-centered approaches that overlook the existence of distinct patterns of social support and thus provide little insight into the joint effects of social support from multiple sources on bullying victims. Using a person-centered approach, this study explored bullying victims’ profiles of perceived social support from five sources and investigated how the profiles of perceived social support correspond with bullying victims’ demographic characteristics, frequency of victimization, psychological health, and prosocial behavior. The results identified five profiles of social support (i.e., *online-offline supported*, *offline supported*, *moderately supported*, *close friend supported*, and *unsupported*). Bullying victims who were younger or from higher-SES families were more likely to fall into the *offline supported* profile than the other four profiles. Girls were more likely to be classified as *online-offline supported*, whereas boys were more likely to be categorised as *moderately supported*. Victims reported more frequent victimization experience were less likely to perceive high social support from multiple sources. Among the five profiles of bullying victims, *offline supported* victims reported optimal outcomes, whereas *close friend supported* and *unsupported* victims showed suboptimal results. These findings support the idea that bullying victims are a heterogeneous group in terms of their social support patterns and underscore high and co-existing support from multiple offline sources for bullying victim’s optimal outcomes.

### Supplementary information

Appendix

## Data Availability

The dataset used in the study is not publicly available but is available from the corresponding author on reasonable request.

## Footnotes

1 Since students in Grade 9 were preparing for the high school entrance examinations, few of them participated in the study.

2 According to the data from the National Bureau Statistics of China (2023), the per capita disposable income of urban residents was 49,283 RMB and the national average number of people per household was 2.62 people per household. Hence, the average monthly family income was approximately 10,760 RMB for urban residents.

## References

[CR1] Agnew R (2007). Pressured into crime: An overview of general strain theory.

[CR2] Allen, K. A., Furlong, M. J., Vella-Brodrick, D., & Suldo, S. M. (Eds.). (2022). *Handbook of positive psychology in schools: Supporting process and practice*. Routledge.

[CR3] Asparouhov T, Muthén B (2014). Auxiliary variables in mixture modeling: Three-step approaches using *Mplus*. Structural Equation Modeling.

[CR4] Asparouhov T, Muthén B (2014). Auxiliary variables in mixture modeling: Using the BCH method in Mplus to estimate a distal outcome model and an arbitrary secondary model. Mplus Web Notes.

[CR5] Boateng GO, Neilands TB, Frongillo EA, Melgar-Quiñonez HR, Young SL (2018). Best practices for developing and validating scales for health, social, and behavioral research: A primer. Frontiers in Public Health.

[CR6] Bollen KA (1989). A new incremental fit index for general structural equation models. Sociological Methods & Research.

[CR7] Boyd DM, Ellison NB (2007). Social network sites: Definition, history, and scholarship. Journal of Computer-Mediated Communication.

[CR8] Bronfenbrenner U (1979). The ecology of human development: Experiments by nature and design.

[CR9] Chan MK, Sharkey JD, Nylund-Gibson K, Dowdy E, Furlong MJ (2022). Social support profiles associations with adolescents’ psychological and academic functioning. Journal of School Psychology.

[CR10] Chiu MM, Chow BW-Y (2015). Classmate characteristics and student achievement in 33 countries: Classmates’ past achievement, family socioeconomic status, educational resources, and attitudes toward reading. Journal of Educational Psychology.

[CR11] Chu JTW, Bullen P, Farruggia SP, Dittman CK, Sanders MR (2015). Parent and adolescent effects of a universal group program for the parenting of adolescents. Prevention Science.

[CR12] Ciarrochi J, Morin AJS, Sahdra BK, Litalien D, Parker PD (2017). A longitudinal person-centered perspective on youth social support: Relations with psychological wellbeing. Developmental Psychology.

[CR13] Cohen, J. (1988). Statistical power analysis for the behavioral sciences (2nd). Hillsdale, NJ: Erlbaum.

[CR14] Conger RD, Donnellan MB (2007). An interactionist perspective on the socioeconomic context of human development. Annual Review of Psychology.

[CR15] Demaray MK, Malecki CK (2002). Critical levels of perceived social support associated with student adjustment. School Psychology Quarterly.

[CR16] Dennehy R, Meaney S, Cronin M, Arensman E (2020). The psychosocial impacts of cybervictimisation and barriers to seeking social support: Young people’s perspectives. Children and Youth Services Review.

[CR17] Desjardins TL, Leadbeater BJ (2011). Relational victimization and depressive symptoms in adolescence: Moderating effects of mother, father, and peer emotional support. Journal of Youth and Adolescence.

[CR18] Diener E, Heintzelman SJ, Kushlev K, Tay L, Wirtz D, Lutes LD, Oishi S (2017). Findings all psychologists should know from the new science on subjective well-being. Canadian Psychology.

[CR19] Duong MT, Pullmann MD, Buntain-Ricklefs J, Lee K, Benjamin KS, Nguyen L, Cook CR (2019). Brief teacher training improves student behavior and student-teacher relationships in middle school. School Psychology.

[CR20] Dvorsky MR, Kofler MJ, Burns GL, Luebbe AM, Garner AA, Jarrett MA, Soto EF, Becker SP (2019). Factor structure and criterion validity of the five Cs model of positive youth development in a multi-university sample of college students. Journal of Youth and Adolescence.

[CR21] García-Vázquez FI, Valdés-Cuervo AA, Martínez-Ferrer B, Parra-Pérez LG (2020). Forgiveness, gratitude, happiness, and prosocial bystander behavior in bullying. Frontiers in Psychology.

[CR22] George, D., & Mallery, P. (2010). SPSS for Windows step by step: A simple study guide and reference (10. Baski). London: Pearson Education.

[CR23] Goodman R (2001). Psychometric properties of the strengths and difficulties questionnaire. Journal of the American Academy of Child and Adolescent Psychiatry.

[CR24] Gower AL, Borowsky IW (2013). Associations between frequency of bullying involvement and adjustment in adolescence. Academic Pediatrics.

[CR25] Greene BA (2015). Measuring cognitive engagement with self-report scales: Reflections from over 20 years of research. Educational Psychologist.

[CR26] Guo J, Li M, Wang X, Ma S, Ma J (2020). Being bullied and depressive symptoms in Chinese high school students: The role of social support. Psychiatry Research.

[CR27] Han X, Han W, Qu J, Li B, Zhu Q (2019). What happens online stays online? Social media dependency, online support behavior and offline effects for LGBT. Computers in Human Behavior.

[CR28] He XY, Li CB, Qian J, Cui HS, Wu WY (2010). Reliability and validity of a generalized anxiety disorder scale in general hospital outpatients. Shanghai Archives of Psychiatry.

[CR29] Hellfeldt K, López-Romero L, Andershed H (2020). Cyberbullying and psychological well-being in young adolescence: the potential protective mediation effects of social support from family, friends, and teachers. International Journal of Environmental Research and Public Health.

[CR30] Holfeld B, Baitz R (2020). The mediating and moderating effects of social support and school climate on the association between cyber victimization and internalizing symptoms. Journal of Youth and Adolescence.

[CR31] Huang L, Yang T, Ji Z (2003). Applicability of the positive and negative affect scale in Chinese. Chinese Mental Health Journal.

[CR32] Humphrey N, Symes W (2010). Responses to bullying and use of social support among pupils with autism spectrum disorders (ASDs) in mainstream schools: A qualitative study. Journal of research in special educational needs.

[CR33] Jiang X, Fang L, Lyons MD (2019). Is life satisfaction an antecedent to coping behaviors for adolescents?. Journal of Youth and Adolescence.

[CR34] Jiang X, Fang L, Stith BR, Liu RD, Huebner ES (2018). A psychometric evaluation of the Chinese version of the students’ life satisfaction scale. Applied Research in Quality of Life.

[CR35] Kong, M., & Lu, Y. (2023). Take You to Know the Tibetan Adolescents: Their Main Social Relationships and the Effect on the Prosocial Behavior. *Children and Youth Services Review*, 107031. 10.1016/j.childyouth.2023.107031.

[CR36] Kroenke K, Spitzer RL, Williams JB (2001). The PHQ‐9: Validity of a brief depression severity measure. Journal of General Internal Medicine.

[CR37] Laursen B, Hoff E (2006). Person-centered and variable-centered approaches to longitudinal data. Merrill-Palmer Quarterly.

[CR38] Lee J, Roh BR, Yang KE (2022). Exploring the association between social support and patterns of bullying victimization among school-aged adolescents. Children and Youth Services Review.

[CR39] Li H, Zhang W, Yu F (2012). The relationship between victimization and depression of adolescents. Psychological Development and Education.

[CR40] Li J, Yao M, Liu H (2021). From social support to adolescents’ subjective well-being: The mediating role of emotion regulation and prosocial behavior and gender difference. Child Indicators Research.

[CR41] Li Q (2007). New bottle but old wine: a research of cyberbullying in schools. Computers in Human Behavior.

[CR42] Lin JH (2016). Need for relatedness: a self-determination approach to examining attachment styles, Facebook use, and psychological well-being. Asian Journal of Communication.

[CR43] Lindow JC, Hughes JL, South C, Minhajuddin A, Gutierrez L, Bannister E, Trivedi MH, Byerly MJ (2020). The youth aware of mental health intervention: Impact on help seeking, mental health knowledge, and stigma in US adolescents. Journal of Adolescent Health.

[CR44] Little RJ, Rubin DB (1989). The analysis of social science data with missing values. Sociological Methods & Research.

[CR45] Longest K, Kang JA (2022). Social media, social support, and mental health of young adults during COVID-19. Frontiers in Communication.

[CR46] Luo S, Chen D, Li C, Lin L, Chen W, Ren Y, Zhang Y, Xing F, Guo VY (2023). Maternal adverse childhood experiences and behavioral problems in preschool offspring: The mediation role of parenting styles. Child and Adolescent Psychiatry and Mental Health.

[CR47] Lyell KM, Coyle S, Malecki CK, Santuzzi AM (2020). Parent and peer social support compensation and internalizing problems in adolescence. Journal of School Psychology.

[CR48] Malecki, C. K., Demaray, M. K., & Elliott, S. N. (2003). *A working manual on the development of the child and adolescent social support scale (2000). Unpublished manuscript*. Northern Illinois University.

[CR49] Massing-Schaffer M, Nesi J, Telzer EH, Lindquist KA, Prinstein MJ (2022). Adolescent peer experiences and prospective suicidal ideation: The protective role of online-only friendships. Journal of Clinical Child & Adolescent Psychology.

[CR50] McDougall P, Vaillancourt T (2015). Long-term adult outcomes of peer victimization in childhood and adolescence: pathways to adjustment and maladjustment. American Psychologist.

[CR51] Morin AJ, Meyer JP, Creusier J, Biétry F (2016). Multiple-group analysis of similarity in latent profile solutions. Organizational Research Methods.

[CR52] Muthén, L. K., & Muthén, B. O. (1998-2017). *Mplus user’s guide (8th ed.)*. Muthén & Muthén.

[CR53] National Bureau Statistics of China. (2023). Households’ income and consumption expenditure in 2022. https://www.stats.gov.cn/sj/zxfb/202302/t20230203_1901715.html.

[CR54] Nesi J, Choukas-Bradley S, Prinstein MJ (2018). Transformation of adolescent peer relations in the social media context: Part 1-a theoretical framework and application to dyadic peer relationships. Clinical Child and Family Psychology Review.

[CR55] Nielsen P, Favez N, Liddle H, Rigter H (2019). Linking parental mediation practices to adolescents’ problematic online screen use: A systematic literature review. Journal of Behavioral Addictions.

[CR56] Nylund KL, Asparouhov T, Muthén BO (2007). Deciding on the number of classes in latent class analysis and growth mixture modeling: A Monte Carlo simulation study. Structural Equation Modeling.

[CR57] Peng, X., Liang, S., Liu, L., Cai, C., Chen, J., Huang, A., Wang, X., & Zhao, J. (2022). Prevalence and associated factors of depression, anxiety and suicidality among Chinese high school E-learning students during the COVID-19 lockdown. *Current Psychology*, 1–12. 10.1007/s12144-021-02512-x. 10.1007/s12144-021-02512-x PMC8791692 35103039

[CR58] Radez, J., Reardon, T., Creswell, C., Orchard, F., & Waite, P. (2021). Adolescents’ perceived barriers and facilitators to seeking and accessing professional help for anxiety and depressive disorders: qualitative interview study. *European Child & Adolescent Psychiatry*, 1–17. 10.1007/s00787-020-01707-0. 10.1007/s00787-020-01707-0 PMC9209355 33502596

[CR59] Rose AJ (2021). The costs and benefits of co‐rumination. Child Development Perspectives.

[CR60] Rueger SY, Malecki CK, Demaray MK (2010). Relationship between multiple sources of perceived social support and psychological and academic adjustment in early adolescence: Comparisons across gender. Journal of Youth and Adolescence.

[CR61] Rueger SY, Malecki CK, Pyun Y, Aycock C, Coyle S (2016). A meta-analytic review of the association between perceived social support and depression in childhood and adolescence. Psychological Bulletin.

[CR62] Salmivalli C, Laninga‐Wijnen L, Malamut ST, Garandeau CF (2021). Bullying prevention in adolescence: solutions and new challenges from the past decade. Journal of Research on Adolescence.

[CR63] Schafer MH, Vargas N (2016). The dynamics of social support inequality: Maintenance gaps by socioeconomic status and race?. Social Forces.

[CR64] Shaffer DR, Kipp K (2014). Developmental Psychology: Childhood and adolescence (9th ed.).

[CR65] Singstad MT, Wallander JL, Greger HK, Lydersen S, Kayed NS (2021). Perceived social support and quality of life among adolescents in residential youth care: A cross-sectional study. Health and Quality of Life Outcomes.

[CR66] Song, R., Chen, L., Zhang, L., Yu, F., & Zhang, W. (2023). Profiles and developmental transitions of educational future orientation among senior high school students in China. *Journal of Youth and Adolescence*, 1–16. 10.1007/s10964-023-01806-6. 10.1007/s10964-023-01806-6 37369926

[CR67] Spitzer RL, Kroenke K, Williams JB, Löwe B (2006). A brief measure for assessing generalized anxiety disorder: The GAD-7. Archives of Internal Medicine.

[CR68] Takano, M., & Yokotani, K. (2022). Online Social Support via Avatar Communication Buffers Harmful Effects of Offline Bullying Victimization. In C. Budak., M. Cha., & D. Quercia (Eds). *Proceedings of the International AAAI Conference on Web and Social Media* (Vol. 16, pp. 980–992). 10.1609/icwsm.v16i1.19351.

[CR69] Tein J-Y, Coxe S, Cham H (2013). Statistical power to detect the correct number of classes in latent profile analysis. Structural Equation Modeling.

[CR70] Tian L, Zhang J, Huebner ES (2015). Preliminary validation of the Brief Multidimensional Students’ Life Satisfaction Scale (BMSLSS) among Chinese elementary school students. Child Indicators Research.

[CR71] Tian L, Tian Q, Huebner ES (2016). School-related social support and adolescents’ school-related subjective well-being: The mediating role of basic psychological needs satisfaction at school. Social Indicators Research.

[CR72] Turanovic JJ, Siennick SE, Lloyd KM (2023). Consequences of victimization on perceived friend support during adolescence. Journal of Youth and Adolescence.

[CR73] Ulmanen S, Soini T, Pietarinen J, Pyhältö K, Rautanen P (2022). Primary and lower secondary school students’ social support profiles and study wellbeing. The Journal of Early Adolescence.

[CR74] Wang J, Iannotti RJ, Nansel TR (2009). School bullying among adolescents in the United States: Physical, verbal, relational, and cyber. Journal of Adolescent Health.

[CR75] Watson D, Clark LA, Tellegen A (1988). Development and validation of brief measures of positive and negative affect: The PANAS scales. Journal of Personality and Social Psychology.

[CR76] Wight RG, Botticello AL, Aneshensel CS (2006). Socioeconomic context, social support, and adolescent mental health: A multilevel investigation. Journal of Youth and Adolescence.

[CR77] Yang H, Yan DM, Li XB, Zhang FF, Ren Y, Wang CY (2015). Application of patient health questionnaire-9 in psychosomatic disease outpatients in a general hospital. Chinese Journal of Behavioral Medicine and Brain Science.

[CR78] Yang L, Xiong Y, Gao T, Li S, Ren P (2023). A person-centered approach to resilience against bullying victimization in adolescence: Predictions from teacher support and peer support. Journal of Affective Disorders.

[CR79] Yeung RS, Leadbeater BJ (2013). Peer victimization and internalizing symptoms from adolescence into young adulthood: Building strength through emotional support. Journal of Research on Adolescence.

[CR80] Zhao Y, Hong JS, Zhao Y, Yang D (2021). Parent-child, teacher-student, and classmate relationships and bullying victimization among adolescents in China: Implications for school mental health. School Mental Health.

[CR81] Zhou J, Luo G (2017). Qingshaonianzaoshouxiaoyuanbalindechengdujiqiyingxiangyinsuyanjiu [A Study on the extent and factors of bullying among adolescents]. Advances in China Public Security.

[CR82] Zhu Z, Ma W, Leng C (2020). ICT adoption, individual income and psychological health of rural farmers in China. Applied Research in Quality of Life.

