# Supplementary material for: Bullying Victims’ Perceived Social Support and Psychological Health and Prosocial Behavior: A Latent Profile Analysis
Source: J Youth Adolesc. 2024 Mar 1;53(7):1683–98. doi: 10.1007/s10964-024-01954-3 (PMC11136783; doi:10.1007/s10964-024-01954-3)

**Appendix A**

Since the *close friend supported* profile is relatively small, sensitivity analysis was conducted to estimate its robustness. In the manuscript, the 5-profile solution was optimal for describing patterns of perceived social support among bullying victims, based on the indices of fit consulted. The 4-profile solution nonetheless demonstrated the highest entropy than the other solutions. Therefore, the profiles identified by the 4-profile solution (Figure A) were presented as well.

**Figure A**

*Standardized Mean Estimates of Profiles Identified by the 4-Profile Solution*


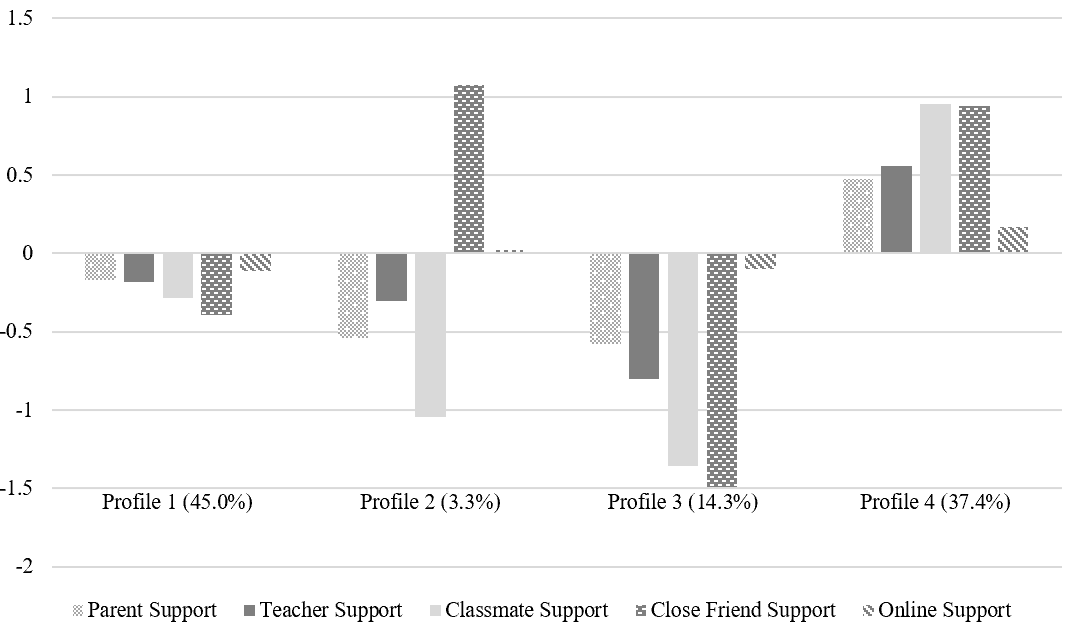

Supplement: Supplementary file 1 — Appendix [file 10964_2024_1954_MOESM1_ESM.docx]
